# Quality of Life Following Salvage Endoscopic Nasopharyngectomy in Patients With Recurrent Nasopharyngeal Carcinoma: A Prospective Study

**DOI:** 10.3389/fonc.2020.00437

**Published:** 2020-04-17

**Authors:** Wanpeng Li, Hanyu Lu, Juan Liu, Quan Liu, Huan Wang, Huankang Zhang, Xicai Sun, Li Hu, Weidong Zhao, Yurong Gu, Houyong Li, Dehui Wang

**Affiliations:** Department of Otolaryngology-Head and Neck Surgery, Affiliated Eye Ear Nose and Throat Hospital, Fudan University, Shanghai, China

**Keywords:** nasopharyngeal carcinoma, recurrent, endoscopic, nasopharyngectomy, quality of life

## Abstract

**Background:** This study aimed to assess the effect of endoscopic nasopharyngectomy in patients with recurrent nasopharyngeal carcinoma (NPC) on site-specific and sinonasal-related quality of life (QoL) before and after surgery using validated instruments.

**Methods:** Consecutive adult patients with recurrent NPC, who were treated via salvage endoscopic nasopharyngectomy, were prospectively enrolled at a single institution from January 2018 to December 2019. Each patient completed the Anterior Skull Base Questionnaire (ASBQ) and the 22-Item Sino-Nasal Outcome Test (SNOT-22) preoperatively, and then at regular intervals after surgery to assess their perceived QoL.

**Results:** Forty patients fulfilled the inclusion criteria. The median follow-up was 12 months (range, 2–24 months). Overall scores on the ASBQ and SNOT-22 at 3 or 12 weeks after surgery decreased significantly compared with before surgery (*p* < 0.05). At 6 months and 1 year postoperatively, there was no significant difference from the preoperative score. Subtotal resection was associated with worse overall ASBQ scores at 6 months and 1 year after endoscopic nasopharyngectomy (*p* < 0.05). Worse QoL was also associated with advanced T stage (rT3 and rT4) and pathological World Health Organization type III. Sex, age (<50 years), tumor necrosis, lymph node metastasis, and use of a nasoseptal flap approach did not impact postoperative QoL.

**Conclusions:** Site-specific and sinonasal-related QoL, measured using validated tools, demonstrated an overall maintenance of postoperative compared with preoperative QoL. Endoscopic endonasal resection is a valuable management choice in patients with recurrent NPC. In addition, subtotal resection was an important factor that negatively influenced postoperative QoL; as such, gross-total resection should be attempted in all patients to optimize QoL after surgery.

## Introduction

Nasopharyngeal carcinoma (NPC) has a high incidence in South China and Southeast Asia, with an obvious ethnic and geographical distribution (1). Radiotherapy is the first choice given the early susceptibility of NPC. However, ~10% of patients experience local recurrence after radiotherapy. Re-irradiation for recurrent NPC may lead to poor curative effect, serious complications, and even death; therefore, surgical treatment of recurrent NPC has been advocated in many studies (2–4). Endoscopic nasopharyngectomy has been increasingly used due to advances in high-magnification technology, and the decreases in functional and cosmetic morbidities (5, 6). Evaluation of the curative effect of endoscopic nasopharyngectomy in the literature has mainly focused on resection, complications, survival rate, and other parameters. However, recent interest in outcome-based studies has led us to recognize the importance of patient perception of their health and the success of surgical interventions. Evaluation of these subjective parameters includes quality of life (QoL) measurements, questionnaires, and direct symptom scores (7).

QoL is a multidimensional metric, which describes an individual's overall perception of happiness. QoL instruments can be generalized, or site- or disease-specific. Data collected directly from patients are referred to as patient-reported outcome measures. Patient perception of QoL is an important indicator to measure the success of surgery, which can be used as an additional outcome measure. It is important to use effective instruments to assess the QoL of distinct sequelae associated with skull base disease and related approaches. In a prospective study involving 66 patients undergoing endoscopic skull base surgery, Edward et al. found that postoperative, site-specific QoL improved compared with the preoperative QoL (7). To our knowledge, no prospective studies have described changes in site-specific QoL in patients undergoing endoscopic nasopharyngectomy for recurrent NPC. Therefore, in this study, we measured postoperative QoL in a series of patients using acceptable site-specific QoL metrics and used their own preoperative QoL as an internal control. Two disease-specific instruments were used: the Anterior Skull Base Questionnaire (ASBQ) and the 22-Item Sinonasal Outcome Test (SNOT-22) (8, 9).

## Methods

### Study Design and Data Sources

Consecutive adult patients with recurrent NPC who were treated via salvage endoscopic nasopharyngectomy were prospectively enrolled at the Department of Otorhinolaryngology of the Affiliated Eye Ear Nose and Throat Hospital (AEENTH) at Fudan University (Shanghai, China) from January 2018 to December 2019. All surgeries were performed by the senior author (DW). The Institutional Review Board of AEENTH at Fudan University approved this study. All patients who underwent endonasal endoscopic surgery for histologically confirmed NPC were identified. Patients selected for inclusion were adults >18 years of age who had completed preoperative assessment with the ASBQ and SNOT-22, and at ≥1 time point postoperatively. Those who required simultaneous neck dissection for metastatic lymph node disease, those who had undergone nasopharyngectomy for other types of malignancy, and those who were unable to complete the questionnaire because of illiteracy were excluded from this study.

The presence of recurrent NPC was detected by routine endoscopy or magnetic resonance imaging (MRI) of the nasopharynx, and confirmed by endoscopic biopsy. The choice of salvage treatment was determined according to tumor location, disease degree, the preferences of patients, and consultation with radiation oncologists and surgeons. All the patients with recurrent NPC were treated via salvage endoscopic nasopharyngectomy. If the tumor was confined to the posterior wall and midline of the nasopharynx, the resection range should reach the basisphenoid superiorly, prevertebral fascia posteriorly, and torus tubarius laterally. When the sphenoid sinus was involved by the tumor, bilateral sphenoidotomies should be performed to remove the floor of the sphenoid sinus, anterior wall, sphenoid rostrum, and intersphenoidal septum. The questionnaires were administered in person or by telephone interview. An independent doctor conducted all interviews to preclude any bias from doctor–patient interaction(s). Patient charts were reviewed for age, sex, tumor necrosis, T stage, lymph node metastasis, extent of resection, and pathological type.

In this study, two effective methods of QoL measurement were used, the ASBQ and SNOT-22. Both questionnaires were completed by each patient before the preoperative evaluation. Postoperative follow-up was performed at 3 weeks, 12 weeks, 6 months, and 1 year after endoscopic nasopharyngectomy, at which time each patient was again asked to complete the ASBQ and SNOT-22. Upon completion of the survey, endoscopic examination and intranasal debridement were performed during each visit. The main outcome measure of this study was postoperative changes in ASBQ scores, followed by postoperative changes in SNOT-22 scores.

### Outcome Measures

The ASBQ consists of 35 items divided into six independent QoL areas: performance, physical function, vitality, pain, emotional impact, and specific symptoms. Responses are scored on a 5-item Likert scale, with each item scored from 1 to 5, and a total score ranging from 35 to 175. The total score is reported as an average item score of 1.0 to 5.0, with a lower score indicating worse QoL. The SNOT-22 questionnaire consists of 22 questions, with responses recorded on a 6-point Likert scale ranging from 0 to 5 points per item. Total scores range from 0 to 110, with a lower score indicating a better QoL.

### Statistical Methods

Statistical analysis was performed using SPSS version 19.0 (IBM Corporation, Armonk, NY, USA). Each case served as its own historical control. The paired *t*-test was used to compare average preoperative and postoperative scores. The correlation between ASBQ and SNOT scores was analyzed by Pearson's correlation. Univariate analysis was performed using the unpaired *t* test. All *P* values were two-sided, and statistical significance was evaluated at the 0.05 alpha level.

## Results

Forty patients fulfilled inclusion criteria for inclusion in the study, 31 (77.5%) of whom were male and 9 (22.5%) were female, with a mean age of 50.7 years (range, 32–69 years). Tumor characteristics of the patients included in this study are summarized in Table 1. All the patients were previously treated with intensity modulated radiotherapy, and 26 patients received concurrently chemotherapy. The median time between initial radiotherapy and recurrence was 36 months (range 6–96 months). Tumor necrosis was observed in 14 (35.0%) patients. Tumors in this study were staged according to the rTNM staging system of the American Joint Committee on Cancer, as follows: rT1 (*n* = 17); rT2 (*n* = 5); rT3 (*n* = 14); and rT4 (*n* = 4). Fifteen (37.5%) patients experienced lymph node metastases. Gross-total resection (GTR) was performed in 30 of 40 operations (75.0%). The histological World Health Organization (WHO) lesion subtype in most patients was type III [*n* = 22 (55.0%)], followed by WHO type II [*n* = 18 (45.0%)]. Skull base defects repaired using a nasoseptal flap was presented in 27 cases (67.5%).

**Table 1:** Summary of tumor characteristics in 40 patients who underwent endoscopic nasopharyngectomy.

**Characteristic**	**Total = 40**	**%**
Sex		
Male	31	77.5
Female	9	22.5
Age		
<50	19	47.5
≥50	21	52.5
Tumor necrosis		
No	26	65.0
Yes	14	35.0
T stage		
T1	17	42.5
T2	5	12.5
T3	14	35
T4	4	10
Lymph node metastasis		
No	25	63.5
Yes	15	37.5
GTR		
No	10	25.0
Yes	30	75.0
Pathological type		
WHO type II	18	45.0
WHO type III	22	55.0
Nasoseptal flap		
NO	13	32.5
Yes	27	67.5

*GTR, Gross-total resection; WHO, World Health Organization*.

**Figure 1:**
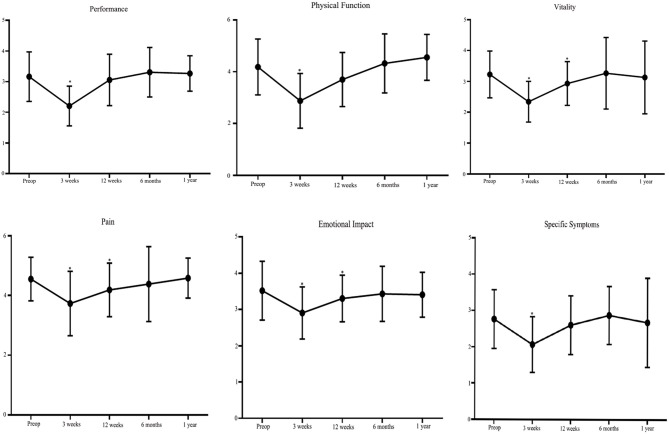
Plots illustrating preoperative and postoperative scores for each of the six domains assessed using the Anterior Skull Base Questionnaire (ASBQ) after endoscopic nasopharyngectomy in patients with recurrent nasopharyngeal carcinoma. A higher score indicates better quality of life. *p < 0.05 (paired *t* test).

Overall ASBQ scores at 3 or 12 weeks after surgery decreased significantly compared with before surgery. At 6 months and 1 year postoperatively, there was no significant difference over the preoperative ASBQ score (Table 2). Independent examination of all subdomains of the ASBQ revealed significant decreases at 3 weeks after surgery, with further decreases in 3 of 6 subdomains (vitality, pain, emotional impact) at 12 weeks postoperatively. Finally, the subdomain of physical function demonstrated improvement at 1 year, while the other 5 subdomains at 6 months and 1 year did not differ significantly after endoscopic nasopharyngectomy (Figure 1).

**Table 2:** Overall postoperative ASBQ scores in patients who underwent endoscopic nasopharyngectomy.

**Time since surgery**	**No. of patients**	**Mean ASBQ score (SD)**		***P* value**
		**Preop**	**Postop**	
3 weeks	32	3.38 (0.60)	2.49 (0.63)	<0.0001*
12 weeks	25	3.43 (0.64)	3.10 (0.68)	0.0444*
6 month	19	3.37 (0.64)	3.42 (0.72)	0.8062
1 year	20	3.22 (0.52)	3.49 (0.57)	0.1686

* *p < 0.05, paired 2-tailed t-test. SD, standard deviation; ASBQ, Anterior Skull Base Questionnaire*.

SNOT-22 scores were higher (i.e., worse outcome) at 3 or 12 weeks after endoscopic nasopharyngectomy compared with preoperative scores. However, subsequent SNOT-22 scores at 6 months and 1 year postoperatively did not differ significantly from preoperative scores (Table 3). Overall, SNOT-22 and ASBQ scores demonstrated a significant inverse correlation preoperatively, and at 3 and 12 weeks postoperatively (*r* = −0.613, *r* = −0.614, *r* = −0.744, respectively) (Table 4). The direction of correlation reflected the inverse direction of scoring for the ASBQ and SNOT-22 questionnaires. Using ASBQ and SNOT-22, we identified four items from each instrument exhibiting the severe symptoms after 1-year follow-up of patients (Table 5). By ASBQ, sense of smell and nasal secretions were the most frequently reported item with high symptom burden (35% of patients reported scores of 1 or 2). Using the SNOT-22 instrument, sense of taste/smell demonstrated the highest symptom burden (40% of patients reported scores of 4 to 6).

**Table 3:** Overall postoperative SNOT-22 scores in patients who underwent endoscopic nasopharyngectomy.

		**Mean SNOT-22 Score (SD)**		
**Time since surgery**	**No. of patients**	**Preop**	**Postop**	***P* value**
3 weeks	32	24.83 (19.71)	45.69 (25.08)	<0.0001*
12 weeks	25	23.64 (19.34)	34.44 (19.75)	0.0006*
6 month	19	22.95 (18.33)	30.26 (17.55)	0.098
1 year	20	24.15 (18.16)	23.95 (9.66)	0.9685

* *p < 0.05, paired 2-tailed t-test.SD, standard deviation; SNOT-22, 22-item Sinonasal Outcome Test*.

**Table 4:** Correlation between SNOT-22 and ASBQ scores.

**Time since surgery**	**ASBQ (n)**	**SNOT-22 (*n*)**	**Pearson correlation coefficient**	***P* value**
Preoperatively	40	40	−0.613	<0.0001*
3 weeks	32	32	−0.614	<0.0001*
12 weeks	25	25	−0.362	0.075
6 month	19	19	−0.744	<0.0001*
1 year	20	19	−0.398	0.082

*ASBQ, Anterior Skull Base Questionnaire; SNOT-22, 22-item Sinonasal Outcome Test*;

* *p < 0.05, Pearson's correlation analysis*.

**Table 5:** The items of ASBQ and SNOT-22 with severe symptoms after 1 year follow-up of patients.

	**ASBQ**		**SNOT-22**	
	**Question/Item**	**Patients (%)**	**Question/Item**	**Patients (%)**
Severe symptoms	“How would you define your sense of smell?	35	“Sense of taste/smell”	40
	“How would you define your amount of nasal secretions”	35	“Blockage/congestion of nose”	35
	“How would you define your sense of taste”	25	“Ear fullness”	30
	“How would you define your eye secretions and tears”	20	“Need to blow nose”	30

*ASBQ, Anterior Skull Base Questionnaire; SNOT-22: 22-Item Sinonasal Outcome Test. Severe symptoms were indicated on ASBQ with scores of 1 or 2 and SNOT-22 with scores of 4 to 6*.

**Figure 2:**
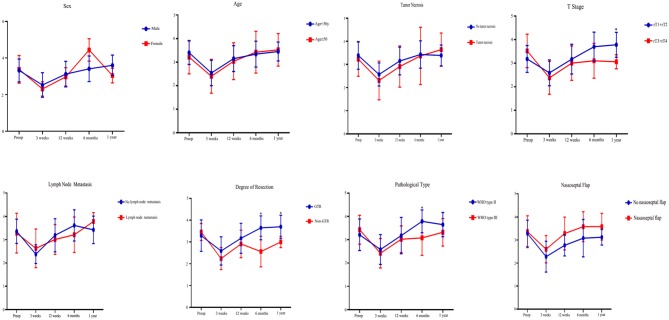
Univariate analysis of Anterior Skull Base Questionnaire (ASBQ) scores for patients who underwent endoscopic nasopharyngectomy for recurrent nasopharyngeal carcinoma. A higher score indicates better quality of life. The *y*-axes represent the scores. *p < 0.05 (two-tailed *t* test).

Univariate analysis was performed for several variables at each postoperative time point (Figure 2). GTR was associated with better overall ASBQ scores and individual domain scores at 6 months and 1 year after endoscopic nasopharyngectomy (*p* < 0.05). rT3 and rT4 lesions adversely affected overall ASBQ scores at 1 year postoperatively. Pathological WHO type III lesions were associated with worse overall ASBQ scores at 6 months postoperatively. There were no significant differences in overall ASBQ scores in terms of sex, age (<50 years), tumor necrosis, lymph node metastasis, or nasoseptal flap approach. A limited number of patients were lost to follow-up, which included 8 patients after the 3-week postoperative visit, 8 patients after the 12-week visit, 7 patients after the 6-month visit, and 5 patients after the 12-month visit. In addition, reasons for loss to follow-up included inability to contact patient (e.g., phone number no longer valid), patient no-shows, and inability of patient to complete visit at each time point because of personal circumstances. Data are being collected for the remaining patients for future analysis.

## Discussion

The assessment of QoL is playing an increasingly important role in evaluating the efficacy of surgery and other substantive factors, such as the extent of resection, incidence of complications, time of progression, and survival rate. Gil et al. were the first to report a QoL study based on a site-specific questionnaire (ASBQ) for patients undergoing anterior skull base surgery. According to the results of their retrospective, cross-sectional study involving 40 patients undergoing open surgery, the authors found that the QoL was generally good, while malignant tumors, comorbidity, and radiotherapy were negative predictors of QoL. However, due to the lack of preoperative data, it was exceedingly difficult to conclude that QoL changes with time (10). The ASBQ was subsequently shown to be psychometrically effective in assessing site-specific QoL following anterior skull base surgery for both endoscopic and transcranial approaches (11, 12). Edward et al. prospectively assessed ASBQ before and after endoscopic skull base surgery in 85 patients and found that overall scores 6 months after surgery demonstrated significant improvement over preoperative scores (13). To avoid bias errors caused by other skull base diseases in the present study, we specifically evaluated changes in ASBQ scores in patients with recurrent NPC before and after endoscopic nasopharyngectomy and found that ASBQ scores at 6 months and 1 year postoperatively were not significantly different over preoperative ASBQ scores. Our results were different from those in some studies describing the assessment of ASBQ changes before and after skull base surgery. The main possible explanation for this difference is that most patients in those studies had benign lesions (7, 13–15).

Postoperative SNOT-22 scores in this study reflected the decrease in early postoperative sinonasal-related QoL, which is consistent with the expected effect of nasal edema, crusting, and nasal secretions after an endonasal surgical approach. These symptoms were usually relieved by postoperative debridement and nasal irrigation, and the SNOT-22 scores had improved to baseline values at 6 months and later time points after surgery. Similar findings were reported in a study in which SNOT-22 scores in 51 patients undergoing endonasal surgery for skull base tumors were significantly higher 6 to 12 months after surgery compared with the first 3 months (11). de Almeida reported that up to 98% of patients who underwent skull base surgery developed nasal crusting that lasted for 100 days (16). In addition, the overall SNOT-22 and ASBQ scores demonstrated a significant inverse correlation, which suggested that assessment using multiple instruments may provide complementary information. It also suggested that a shorter and more concise instrument may be developed to replace the two instruments that cover related domains.

In the literature, only a few studies have investigated the QoL of patients who experienced recurrent NPC after salvage surgery (17, 18). In a prospective, longitudinal study assessing the QoL of 185 patients who underwent nasopharyngectomy using the maxillary swing approach, Chan et al. reported that postoperative QoL of patients was good. However, the most common complications after surgery were trismus, palatal fistula, osteoradionecrosis, maxillary necrosis, and facial numbness and scar(s) (18). In addition, You et al. retrospectively assessed the QoL of patients with T1–T3 recurrent NPC who survived > 3 years after endoscopic nasopharyngectomy or intensity-modulated re-irradiation alone, they found that endoscopic nasopharyngectomy may be a more promising salvage treatment to maximize QoL benefits than re-irradiation (2). To our knowledge, there have been no prospective studies investigating the QoL of patients with recurrent NPC undergoing endoscopic salvage surgery. Results of the present prospective study revealed that short-term QoL after surgery was worse than before surgery; however, long-term QoL after surgery was essentially unchanged. Endoscopic surgery uses the natural passage of the nasal cavity to enter the pathological area of the nasopharynx, which shortens the operation path and reduces damage to the surrounding non-surgical area. In addition, it has the advantages of good illumination, clear vision, no facial incision, and rapid postoperative recovery. Endoscopic surgery can reach almost the same resection range as open surgery, and the QoL of patients after surgery is significantly improved (19, 20).

GTR was achieved using endoscopic surgery in most patients in this series, which resulted in better postoperative QoL than in patients who underwent subtotal resection, which may reflect a favorable sense of well-being by the patient who considers the surgery to be a success. The risk for local tumor recurrence is significantly lower in patients who undergo GTR compared with those who undergo subtotal resection (21). Achieving GTR in recurrent NPC is challenging due to the high incidence of submucosal extension beyond the boundary of the ulceration and the unique tumor configuration (22). Chan and Wei reported that 15-mm radial mucosal margins with the entire medial pterygoid muscle as the deep margin was adopted to ensure an acceptably high probability of achieving GTR during nasopharyngectomy (21). Meanwhile, some studies also reported that skull base tumor, such as pituitary adenomas and craniopharyngiomas, demonstrated a better QoL score after GTR (7, 14, 15).

Several limitations to this study merit consideration. First, a small sample size with long-term QoL data may have led to insufficient detection of some actual differences between QoL before and after surgery. Second, complications have a significant impact on QoL; however, we did not use this variable to stratify QoL due to the small number of complications. Third, surveys used to assess QoL are inherently limited because they may not capture disease-specific QoL changes caused by recurrent NPC and may reflect comorbidities. Finally, the outcomes in this study were based on observational data and, as such, the possibility of confounding factors cannot be excluded, such as socioeconomic status and educational level. However, the low variability in the observed preoperative and postoperative data points provides reassurance about the validity of QOL changes.

## Conclusion

We report the first prospective study investigating site-specific and sinonasal-related QoL after endoscopic nasopharyngectomy in patients who experienced recurrent NPC. Validated QoL tools demonstrated an overall maintenance of postoperative compared with preoperative QoL. Therefore, endoscopic endonasal resection appeared to be a valuable management choice in patients with recurrent NPC. In addition, subtotal resection was an important factor that negatively influenced postoperative QoL; as such, GTR should be attempted in all patients to optimize QoL after surgery.

## Data Availability Statement

The raw data supporting the conclusions of this article will be made available by the authors, without undue reservation, to any qualified researcher.

## Ethics Statement

The studies involving humans were reviewed and approved by the Institutional Review Board of AEENTH at Fudan University. The patients/participants provided their written informed consent to participate in this study.

## Author Contributions

DW and WL conceived and designed the study. WL, HLu, JL, QL, HW, and HZ acquired the data. WL, XS, LH, WZ, YG, and HLi drafted the manuscript. HLu performed the statistical analysis. DW supervised the study.

## Conflict of Interest

The authors declare that the research was conducted in the absence of any commercial or financial relationships that could be construed as a potential conflict of interest.
